# Application of circulating tumor DNA in prospective clinical oncology trials – standardization of preanalytical conditions

**DOI:** 10.1002/1878-0261.12037

**Published:** 2017-02-22

**Authors:** Lisanne F. van Dessel, Nick Beije, Jean C.A. Helmijr, Silvia R. Vitale, Jaco Kraan, Maxime P. Look, Ronald de Wit, Stefan Sleijfer, Maurice P.H.M. Jansen, John W.M. Martens, Martijn P. Lolkema

**Affiliations:** ^1^ Department of Medical Oncology Erasmus MC Cancer Institute Erasmus University Medical Center Rotterdam The Netherlands; ^2^ Workgroup Cancer Genomics Netherlands Erasmus MC Cancer Institute Erasmus University Medical Center Rotterdam The Netherlands

**Keywords:** blood collection tube, cell‐free DNA, circulating tumor DNA, DNA contamination, preanalytical condition

## Abstract

Circulating tumor DNA (ctDNA) has emerged as a potential new biomarker with diagnostic, predictive, and prognostic applications for various solid tumor types. Before beginning large prospective clinical trials to prove the added value of utilizing ctDNA in clinical practice, it is essential to investigate the effects of various preanalytical conditions on the quality of cell‐free DNA (cfDNA) in general and of ctDNA in particular in order to optimize and standardize these conditions. Whole blood samples were collected from patients with metastatic cancer bearing a known somatic variant. The following preanalytical conditions were investigated: (a) different time intervals to plasma isolation (1, 24, and 96 h) and (b) different preservatives in blood collection tubes (EDTA, CellSave, and BCT). The quality of cfDNA/ctDNA was assessed by DNA quantification, digital polymerase chain reaction (dPCR) for somatic variant detection and a β‐actin fragmentation assay for DNA contamination from lysed leukocytes. In 11 (69%) of our 16 patients, we were able to detect the known somatic variant in ctDNA. We observed a time‐dependent increase in cfDNA concentrations in EDTA tubes, which was positively correlated with an increase in wild‐type copy numbers and large DNA fragments (> 420 bp). Using different preservatives did not affect somatic variant detection ability, but did stabilize cfDNA concentrations over time. Variant allele frequency was affected by fluctuations in cfDNA concentration only in EDTA tubes at 96 h. Both CellSave and BCT tubes ensured optimal ctDNA quality in plasma processed within 96 h after blood collection for downstream somatic variant detection by dPCR.

**Abbreviations**
cfDNA: cell‐free DNA
ctDNA: circulating tumor DNA
dPCR: digital polymerase chain reaction
EDTA: ethylenediaminetetraacetic acid
IQR: interquartile ranges
VAF: variant allele frequency

## 1 Introduction

Circulating tumor DNA (ctDNA) has emerged as a potential new biomarker in the field of oncology. The quantification and characterization of ctDNA in plasma creates numerous potential applications, including detection of minimal residual disease, early evaluation of treatment response, and stratification for targeted therapy according to specific genetic changes (Bidard *et al*., 2014; Dawson *et al*., 2013; Diaz and Bardelli, 2014; Diehl *et al*., 2008; Forshew *et al*., 2012; Murtaza *et al*., 2013; Shinozaki *et al*., 2007).

The application of ctDNA‐based diagnostic tests into the clinic still faces several technical difficulties. The biggest hurdle might be the detection limit: ctDNA may comprise < 1.0% of the total cell‐free DNA (cfDNA), making detection of the tumor‐specific fraction challenging (Diehl *et al*., 2005, 2008; Holdhoff *et al*., 2009). The majority of cfDNA is derived from apoptotic tissue and hematological cells which release their DNA in the circulation (Elshimali *et al*., 2013; Jahr *et al*., 2001). Thus, the absolute quantity of cfDNA (‘the background’) determines our ability to detect ctDNA, and quantification of the tumor‐specific variant frequency depends both on the abundance of ctDNA molecules and on the total amount of cfDNA. One of the most important factors impacting the total amount of cfDNA is the time to plasma processing after blood collection, which increases the release of wild‐type DNA from lysed hematological cells present in the blood collection tube (Norton *et al*., 2013; Xue *et al*., 2009). To avoid this, plasma needs to be separated from the blood sample within hours after the blood draw, but the maximum time frame to do so, remains to be revealed.

Due to logistical and practical reasons, it is often not possible to process and store blood samples immediately after blood withdrawal to ensure optimal ctDNA quality; especially in the context of large multicenter prospective clinical trials, which are essential to definitely establish ctDNA as a clinically relevant new biomarker, there is a need for standardization of preanalytical conditions that allow longer processing time of blood samples. To overcome this problem, specialized ‘cell‐stabilizing’ blood collection tubes have been developed. These tubes should not only minimize contamination by wild‐type DNA from lysed hematological cells in the blood tube, but also preserve the quality of ctDNA for reliable downstream analyses.

Until today, a number of studies have tested the different available blood collection tubes to optimally preserve cfDNA/ctDNA (Norton *et al*., 2013; Rothwell *et al*., 2016; Sherwood *et al*., 2016; Toro *et al*., 2015). They all demonstrate a time‐dependent increase in cfDNA concentrations in ethylenediaminetetraacetic acid (EDTA) tubes, while cfDNA concentrations remained stable in both BCT and CellSave tubes. Toro *et al*. (2015) included the PAXgene blood DNA tube in their study, but this tube did not improve the results obtained with EDTA tubes. Yet, even though preservation methods have been compared (Kang *et al*., 2016), thorough direct comparisons between BCT and CellSave tubes at clinically relevant time frames are missing. We set out to compare the available preservatives to allow easier implementation of ctDNA‐based tests into larger clinical trials where processing of samples within 1 h presents a major logistical challenge. The purpose of this study was to investigate the effect on the quality of cfDNA in general and of ctDNA in particular in patients with metastatic cancer under the following preanalytical conditions: (a) different time intervals to plasma isolation (1, 24, and 96 h) and (b) different types of preservative in the blood collection tubes (EDTA, CellSave, and BCT tubes). To this purpose, the amount of cfDNA isolated from plasma was quantitated, its size determined, and the fraction of ctDNA determined.

## 2 Materials and methods

### 2.1 Patient characteristics and somatic variant status of tumor

Between October 2015 and January 2016, cancer patients within the Erasmus MC Cancer Institute in Rotterdam, the Netherlands, were invited to contribute blood samples for this study by their treating physician. Patients were included if they had metastatic disease, were not currently receiving systemic treatment, and if a validated digital polymerase chain reaction (dPCR) assay (TaqMan^®^ SNP genotyping assays, ThermoFisher Scientific, Waltham, MA USA; see also section 2.4) was available for the known somatic variant in their primary and/or metastatic lesion. Somatic variant status and variant allele frequency (VAF) in tissue had been assessed as part of the standard of care by the molecular diagnostics laboratory of the department of pathology in the Rotterdam region by either Sanger sequencing (patient #10 and #16), SNaPshot analysis (patient #05), or NGS analysis (all other patients). The DNA input for these analyses ranged from 0.48 to 10 ng. The calculation of VAF was made through NGS analysis by calculating the coverage of the variant nucleotide relative to the total coverage on that position. For tissue samples analyzed by Sanger sequencing, the VAF was calculated by determining the ratio between the variant peak and the wild‐type peak. All patients provided written informed consent, and the institutional review board approved the protocols (Erasmus MC ID MEC 15‐616).

### 2.2 Preanalytical conditions

After obtaining written informed consent, 9 × 10 mL of blood samples was collected within a single blood draw (see Fig. S1). Matched blood samples were collected in sterile 3 × 10 mL K_2_EDTA tubes (ETDA) (BD Vacutainer^®^, Becton Dickinson, Franklin Lanes, NJ, USA), 3 × 10 mL Cell‐Free DNA™ BCT (BCT) (Streck Inc., Omaha, NE, USA), and 3 × 10 mL CellSave Preservative tubes (Janssen Diagnostics, Raritan, NJ, USA) according to the manufacturer's instructions. The blood samples from one of each type of tube (EDTA, BCT, and CellSave) were processed for plasma isolation at three different time points: within 1 h, at 24 h and at 96 h after blood draw (see Fig. S1). Plasma was isolated using two sequential centrifugation steps: (a) 1711 ***g*** for 10 min at room temperature and (b) 12 000 ***g*** for 10 min at room temperature. Plasma was stored at −80 °C in 1 mL aliquots immediately after centrifugation until further processing.

### 2.3 cfDNA isolation and quantification

For cfDNA isolation, plasma samples were thawed at 4 °C and 3 mL of plasma per sample was used. cfDNA was isolated using the QIAamp^®^ Circulating Nucleic Acid kit (QIAGEN, Venlo, Limburg, The Netherlands) according to the manufacturer's instructions. cfDNA was eluted from the QIAGEN^®^ Mini column using 50 μL buffer AVE which was applied three times to the column to obtain the highest cfDNA concentration possible. cfDNA was stored at −20 °C. cfDNA concentrations were quantified using the Quant‐iT dsDNA high‐sensitivity assay (Invitrogen, Life Technologies, Carlsbad, CA, USA) according to the manufacturer's instructions, and the Qubit fluorometer (Invitrogen) was used as readout.

### 2.4 Digital PCR TaqMan^®^ SNP genotyping and β‐actin fragmentation assay

Cell‐free DNA samples were thawed at room temperature. Validated TaqMan^®^ SNP genotyping assays (ThermoFisher Scientific) were used for somatic variant and wild‐type detection according to the manufacturer's instructions (see Table S1). Accordingly, the limit of detection of this assay is 0.1% (ThermoFisher Scientific, 2016). The maximum volume input of 7.8 μL of the final cfDNA eluate was used, unless the amount of cfDNA in this volume exceeded the maximal input of 30 ng cfDNA, and then 30 ng cfDNA was used. Depending on the obtained cfDNA concentration after plasma isolation, at least 2.57 ng cfDNA was analyzed, leading to a limit of detection of 0.78%.

The TaqMan^®^ β‐actin fragmentation assay was based on the assay developed by Norton *et al*. (2013) to detect a small (136‐bp) and long (420‐bp) β‐actin fragments. We adapted the assay so that both fragments were measured within a single experiment using the reported primers, but different probes for each fragment (see Table S2). For the β‐actin fragmentation assay, a standardized input of 2 ng cfDNA was used to minimize the change of having multiple DNA fragments in one well.

All dPCRs were performed with the QuantStudio 3D dPCR System (ThermoFisher Scientific) according to the manufacturer's protocol. In short, dPCR reaction mix was prepared containing 8.7 μL QuantStudio 3D dPCR Master Mix v2, 0.44 μL Taqman primer/probe mix, up to 7.8 μL of cfDNA, and the total volume was completed with PCR‐grade H_2_O to a final volume of 17.4 μL. Using the QuantStudio 3D dPCR Chip Loader, samples were partitioned on a 20 000‐well QuantStudio 3D dPCR Chip v2 followed by a PCR on a ProFlex 2x Flat PCR System with the following program: 10 min at 96 °C, 40× cycles of 2 min at 60 °C, followed by 30 s at 98 °C, 2 min at 60 °C, and pause at 10 °C. The dPCR data were then acquired with the QuantStudio 3D dPCR Instrument, and the data were analyzed with the QuantStudio 3D Analysis Suite by one technician (JH) to account for interobserver variability.

### 2.5 Statistical analysis

The Wilcoxon signed rank test was used to compare the difference between matched 1‐h and 24‐h samples relative to the difference between matched 1‐h and 96‐h samples. The Friedman test was used to test the order of the three 1‐h samples. To correct for multiple testing, we adjusted the *P* value for significance using the Bonferroni correction. Significance was thus defined as *P* < 0.008 (0.05/6). Correlations were tested by Spearman's rank correlation coefficient.

Cell‐free DNA concentrations determined by the Quant‐iT dsDNA high‐sensitivity assay were corrected for the plasma input and were converted from ng per mL plasma to copies per mL plasma by taking into consideration that 3.3 pg of human DNA contains one copy of a single gene. cfDNA concentrations were then log‐transformed.

To correct for differences in plasma input used for cfDNA isolation and for differences in elution volume after cfDNA isolation, we expressed dPCR results as variant/wild‐type copies per mL plasma. To calculate variant/wild‐type copies per mL plasma, the following equation as described by Lo *et al*. (1998) was used:

$$C=Q\times \left(V_{\text{DNA}}\right)/V_{\text{PCR}})\times \left(1/V_{\text{ext}}\right),$$

where *C* is variant/wild‐type copies per mL plasma; *Q* is the total number of variant/wild‐type copies determined by dPCR; *V*_*DNA*_ is the total volume of cfDNA obtained after cfDNA isolation; *V*_*PCR*_ is the volume of cfDNA solution used for the dPCR reaction; and *V*_*ext*_ is the volume of plasma used for cfDNA isolation.

To calculate VAF, we divided the variant copies per mL plasma by the sum of variant and wild‐type copies per mL plasma.

All statistical analyses were performed using stata version 14.1 (StataCorp LLC, College Station, TX, USA). All figures were plotted using r version 3.2.3 (R Foundation for Statistical Computing, Vienna, Austria).

## 3 Results

### 3.1 Somatic variant detection rate in ctDNA of recruited patients

A total of 16 patients were included who all met the set criteria to investigate the effect of different preanalytical conditions on the quality of ctDNA. Somatic variant status of the primary and/or metastatic lesion had been previously assessed, either by targeted next‐generation sequencing (13 of 16 patients), by SNaPshot analysis (one of 16 patients), or by traditional Sanger sequencing (two of 16 patients). Table 1 lists the origin of the primary tumor, the site and number of metastases, and the VAF in the tumor tissue. Using the specific TaqMan SNP genotyping assay (see Table S1), we were able to detect in 11 (69%) of our 16 patients the known somatic variant in ctDNA isolated within 1 h from EDTA tubes. This corresponds to the detection of 13 (68%) of 19 of the total number of somatic variants tested as some patients had multiple known somatic variants.

**Table 1:** Tumor characteristics and somatic variant detection

Patient ID (#)	Primary tumor	Site and number of metastases (*x*)	Interval tumor tissue and plasma analysis (months)	Known somatic variant (nucleotide change)	VAF in tissue (%)	VAF in plasma EDTA 1 h (%)	cfDNA concentration in plasma EDTA 1 h (copies per mL plasma)
01	Cholangiocarcinoma	Li (3), Lu (2), LN (1)	2	KRAS p.G12D (c.35G>A)	40	0.00	3655
02	Pancreatic cancer	Li (3), Lu (1) LN (6)	9	KRAS p.G12V (c.35G>T)	62	0.00	4055
	CRC			BRAF p.V600E (c.1799T>A)	39	0.97	
				PIK3CA p.H1047R (c.3140A>G)	38	1.86	
03	Breast cancer	LN (> 2)	−1^a^	PIK3CA p.H1047L (c.3140A>T)	26	0.00	2788
04	Melanoma	Li (2), LN (5)	2	BRAF p.V600E (c.1799T>A)	3	1.44	1615
05	CRC	Li (6), LN (2)	6	KRAS p.G13D (c.38G>A)	Unknown	65.46	223 130
06	CRC	Li (3), Lu (4)	18	KRAS p.G12D (c.35G>A)	44	8.61	2215
07	Melanoma	Brain (2), Abd (7)	8	NRAS p.Q61R (c.182A>G)	68	17.22	4245
08	Melanoma	LN (3), Lu (6), Li (> 15), spleen (1), bone (4), peritonitis carcinomatosa, pleuritis carcinomatosa	1	BRAF p.V600E (c.1799T>A)	64	37.21	22 442
09	Melanoma	LN (5)	1	BRAF p.V600E (c.1799T>A)	70	6.42	2739
10	CRC	Brain (2), Li (1), Lu (8)	87	KRAS p.G13D (c.38G>A)	50	0.00	6030
11	CRC	Lu (2)	5	KRAS p.G13D (c.38G>A)	57	0.84	4670
12	CRC	Li (> 20), LN (1)	3	KRAS p.Q61R (c.182A>G)	46	0.00	16 136
13	NSCLC	Brain (8), adrenal gland (1)	7	EGFR p.T790M (c.2369C>T)	17	1.18	5358
				EGFR p.L858R (c.2573T>G)	17	2.62	
14	Melanoma	LN (7), Lu (5), adnexa	22	BRAF p.V600E (c.1799T>A)	56	5.37	3539
15	NSCLC	Li (unknown)	1	EGFR p.T790M (c.2369C>T)	65	27.60	14 085
16	Melanoma	Brain (1)	38	BRAF p.V600E (c.1799T>A)	> 50	0.00	3012

a A new biopsy was taken 2.5 weeks after the blood collection.

CRC, colorectal cancer; NSCLC, non‐small‐cell lung cancer; Li, liver; Lu, lung; LN, lymph node; Abd, abdomen.

### 3.2 Temporal effect of storage in EDTA tubes on cfDNA quality

**Figure 1:**
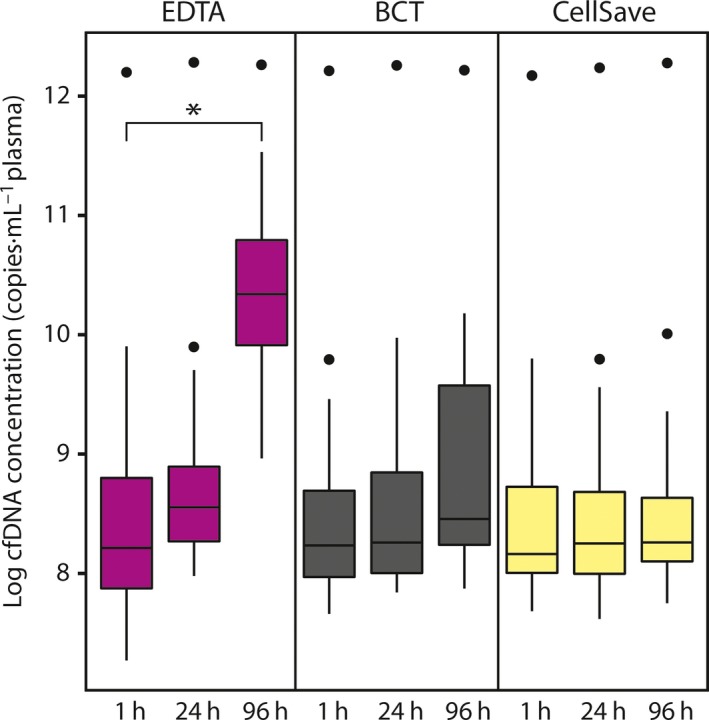
cfDNA concentrations for different preanalytical conditions. Boxes [interquartile ranges (IQR)] and whiskers (1.5 × IQR) are shown together with the median (black horizontal line) of the log cfDNA concentrations in copies per mL plasma of 16 patients for the different preanalytical conditions. Outliers are displayed as black dots. The Wilcoxon signed rank test was used to compare the difference between matched 1‐h and 24‐h samples relative to the difference between matched 1‐h and 96‐h samples. *P < 0.001.

**Figure 2:**
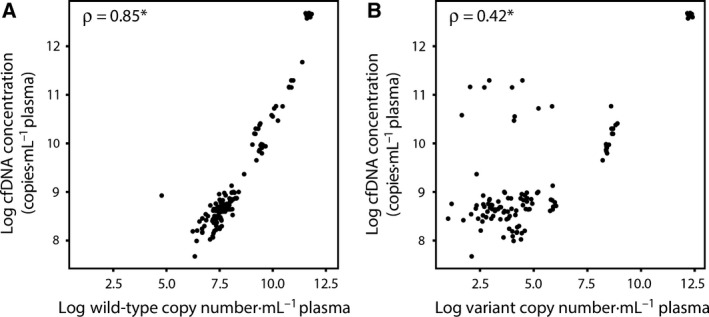
Correlation between wild‐type or variant copy numbers and cfDNA concentration. The log number of wild‐type copies (A) or variant copies (B) in copies per mL plasma on the *x*‐axis is plotted against the log cfDNA concentrations in copies per mL plasma on the *y*‐axis. Data points correspond to single sample measurements from each time interval and each type of preservative. Correlations were tested by Spearman's rank correlation coefficient. *P < 0.001. Five patients with undetectable variant copy numbers in ctDNA are removed from plot B.

To investigate the effect of different time intervals from blood withdrawal to plasma isolation on cfDNA quality, we measured cfDNA concentration isolated from plasma collected in EDTA tubes. We observed a significant increase in cfDNA concentrations in samples isolated after 96 h compared to samples isolated within 1 h (*P* < 0.001; see Fig. 1 and Fig. S2). This increase in cfDNA concentration was significantly positively correlated with an increase in wild‐type copy numbers (rho = 0.85; *P* < 0.001; see Fig. 2A). If a somatic variant was detected in the 1‐h sample, the somatic variant could also be detected in 24 and 96‐h samples. We also observed a significant positive correlation between variant copy numbers and cfDNA concentration, although this was less strong (rho = 0.42; *P* < 0.001; see Fig. 2B).

**Figure 3:**
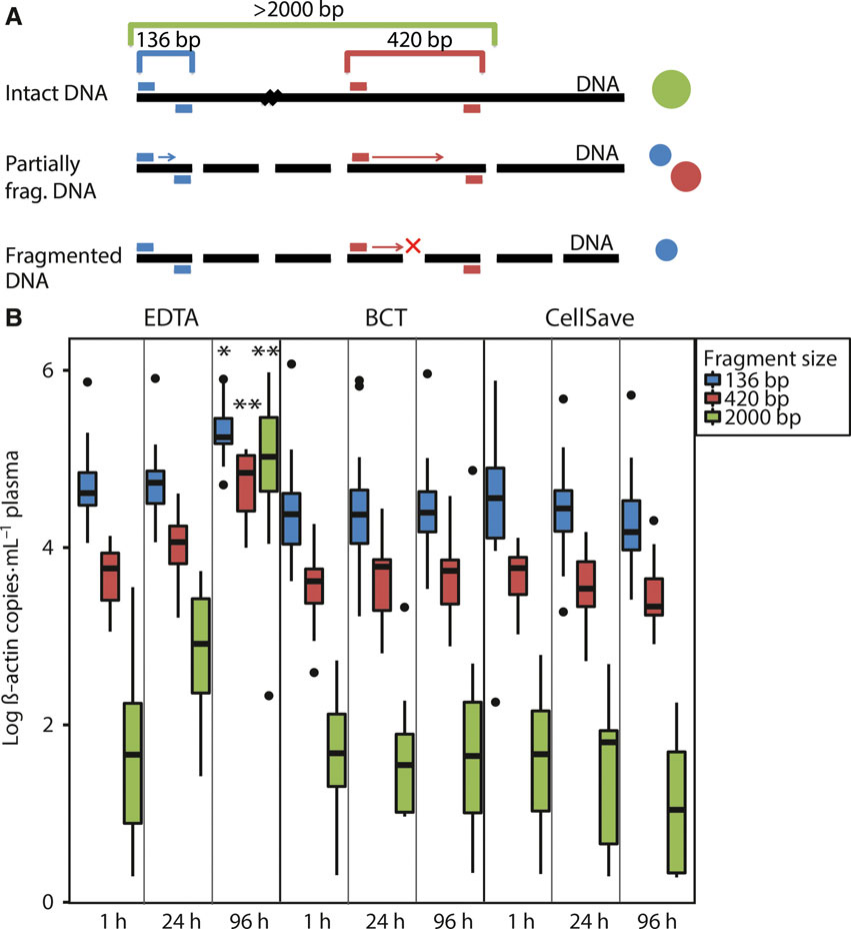
β‐Actin fragmentation assay for different preanalytical conditions. (A) Principle of β‐actin fragmentation assay. dPCR wells containing only 136‐bp signal are indicative of fragmented DNA (fragments < 200 bp), whereas the 420‐bp primer set will only bind to intact DNA (> 420 bp). When a large intact DNA fragment (> 2000 bp) is present in one of the wells, both primer sets can bind, resulting in a mixed signal. In theory, this can also occur when a small (< 200 bp) and large (> 420 bp) DNA fragment is present together in one well.(B) Results of β‐actin fragmentation assay. Boxes [interquartile ranges (IQR)] and whiskers (1.5 × IQR) are shown together with the median (black horizontal line) of the number of β‐actin copies for the different preanalytical conditions. Outliers are displayed as black points. The Wilcoxon signed rank test was used to compare the difference between matched 1‐h and 24‐h samples relative to the difference between matched 1‐h and 96‐h samples for the different fragment sizes. *P = 0.002; **P < 0.001.

To investigate whether the increase in cfDNA concentrations and wild‐type copy numbers was due to the release of intact DNA from lysed leukocytes, we used the β‐actin fragmentation assay (see Fig. 3A). In all preanalytical conditions, we detected low amounts of large fragments. We observed significantly more large fragments in samples from 96 h than in samples from 1 h (420 bp *P* < 0.001; 2000 bp *P* < 0.001; see Fig. 3C). There was also a small but significant increase in fragmented DNA in samples from 96 h compared to samples from 1 h (136 bp *P* = 0.002; Fig. 3B).

### 3.3 The interaction between different preservatives and plasma isolation time intervals and cfDNA quality

Next, we studied the effect of different preservatives in blood collection tubes on cfDNA quality. We compared cfDNA concentrations isolated from plasma collected in EDTA, BCT, and CellSave tubes processed within 1 h. Cell‐free DNA concentrations were similar in all blood collection tubes (see Fig. 1 and Fig. S2). We also did not observe any differences in the DNA fragment size distribution with the β‐actin fragmentation assay for the different tubes at 1 h (see Fig. 3B).

In order to investigate whether the used preservatives in BCT and CellSave tubes could prevent the time‐dependent increase in cfDNA concentration observed in EDTA tubes, we measured cfDNA concentrations in samples isolated after 24 h and 96 h after blood withdrawal. We observed stable cfDNA concentrations in all 24‐h and 96‐h samples compared to their matched 1‐h samples (see Fig. 1 and Fig. S2). Also, we did not observe any differences in the DNA size distribution with the β‐actin fragmentation assay for the matched time intervals for both tube types (see Fig. 3B).

### 3.4 The interaction between different preservatives and plasma isolation time intervals on somatic variant detection in ctDNA

**Figure 4:**
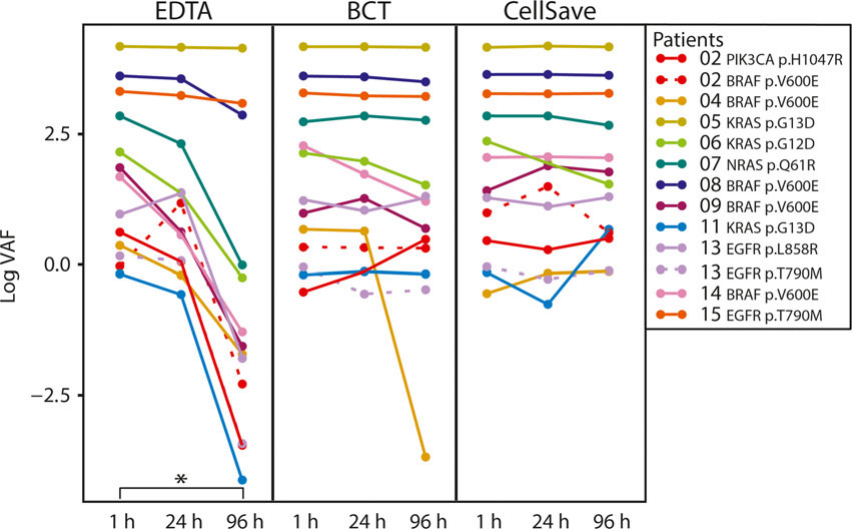
VAF of 11 patients for different preanalytical conditions. Data points correspond to VAF for each individual patient and assay. The Wilcoxon signed rank test was used to compare the difference between matched 1‐h and 24‐h samples relative to the difference between matched 1‐h and 96‐h samples. *P = 0.003.

**Figure 5:**
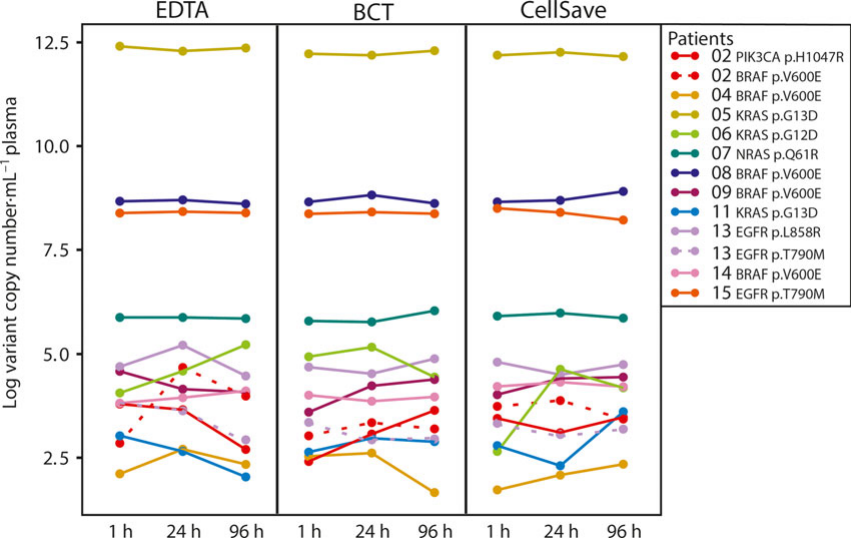
Variant copy numbers of 11 patients for different preanalytical conditions. Data points correspond to variant copy numbers for each individual patient and assay. The Wilcoxon signed rank test was used to compare the difference between matched 1‐h and 24‐h samples relative to the difference between matched 1‐h and 96‐h samples.

To study the effect of time‐dependent increase in cfDNA concentrations and wild‐type copy numbers on somatic variant detection, we analyzed VAF in the different preanalytical conditions compared to their matched 1‐h sample. If a somatic variant was detected in the EDTA 1‐h sample, the somatic variant could also be detected in all BCT and CellSave samples. There was no correlation between the VAF in tumor tissue and the VAF in plasma (see Fig. S3). There was a significant decrease in VAFs in samples from EDTA 96 h (*P* = 0.003; see Fig. 4), which was not observed for the other preanalytical conditions. Because all tubes were drawn within a single blood draw, we expected, in contrast to VAF, that all tubes within each patient contain similar amounts of variant copy numbers. Indeed, variant copy numbers appeared largely similar between tubes and in all tubes compared to their matched 1‐h sample (see Fig. 5 and Fig. S4).

## 4 Discussion and conclusions

The purpose of this study was to investigate the effects of various preanalytical conditions on the quality of cfDNA in general and of ctDNA in particular. The main aim was to investigate whether BCT and CellSave tubes processed within 96 h after blood withdrawal into plasma were suitable for downstream analyses of ctDNA.

Patients were recruited with a high prior probability to harbor ctDNA in their plasma, that is, patients with metastatic disease without current anticancer treatment. In 69% of our patients, we were able to detect the known somatic variant from tissue in ctDNA and this corresponds to the detection of 68% of all tested somatic variants. In two of six missed somatic variants, the somatic variant status in tissue was assessed > 3 years ago. It may be possible that other cancer subclones have emerged, resulting in undetectable somatic variants in ctDNA. Unfortunately, in these cases, more recent information on somatic variant status was not available. Detection of somatic variants in plasma may also be influenced by the site and extent of metastases, which is exemplified by patient #05. This patient had a widespread pattern of metastases with corresponding high levels of cfDNA and high levels of variant copy numbers in plasma. However, due to our heterogeneous cohort, this relationship could not be tested statistically for the other patients.

The clinical utility and potential importance of our methods is evidenced by our findings in patient #02, who was thought to have metastases from his pancreatic carcinoma (first primary cancer) harboring a *KRAS* mutation. However, we could only detect *BRAF* and *PIK3CA* mutations in his ctDNA, highly suggestive that the metastases were originating from the patients’ colorectal cancer (second primary cancer), which can have important implications for his disease management.

The formation of small DNA fragments (180–200 bp lengths) is a biochemical hallmark of apoptosis, whereas during cell lysis or necrosis intact genomic DNA and thus much larger DNA fragments (50–300 kbp) remain (Bortner *et al*., 1995). Through an increase in wild‐type copy numbers and mainly intact DNA fragments, we were able to demonstrate that the time‐dependent increase in cfDNA concentration in EDTA tubes indeed originates from leukocyte lysis. In addition, we observed low levels of intact DNA fragments in all preanalytical conditions, indicating a background level of leukocyte lysis. Both Norton *et al*. (2013) and Rothwell *et al*. (2016) observed a similar increase in cfDNA concentrations in samples collected in EDTA tubes. In both BCT and CellSave tubes, cfDNA concentrations, wild‐type copy numbers, and β‐actin fragment sizes remained stable up to 96 h, indicating that the preservative in these tubes does not adversely affect cfDNA quality. Interestingly, there was also a significant increase in fragmented DNA in samples from EDTA 96 h, which might be attributed to nucleases remaining active.

As we only used dPCR for downstream analysis of ctDNA, we cannot rule out the possibility that the used preservatives in BCT and CellSave tubes could potentially damage the cfDNA and thus affect other downstream analyses. Rothwell *et al*. (2016) assessed the number of single nucleotide variants through whole‐genome sequencing of cfDNA isolated from plasma collected in CellSave tubes. They did not observe introduction of DNA errors. Thus, the preservative used in CellSave tubes does not seem to influence cfDNA downstream analysis using NGS.

Despite the contamination with intact cfDNA, we were still able to detect all somatic variants in ctDNA from EDTA 96‐h samples, in those samples where we were able to detect a somatic variant in the EDTA 1‐h samples. These data suggest that stored samples which have not been processed optimally for ctDNA analysis can still be used to determine the presence of somatic variants in ctDNA. As a consequence of increased cfDNA concentrations and correlated wild‐type copy numbers, we did observe a significant decrease in VAF in the EDTA 96‐h samples. With respect to ctDNA applications for treatment response evaluation, this could result in serious misinterpretations of VAFs. However, variant copy numbers remained stable in all tubes and might thus be a more accurate outcome measure to evaluate treatment response in patients with cancer. Further investigation is needed to determine the interassay variability regarding the range of variant copy numbers and VAFs we observed among the different tubes.

The results in this study indicate that EDTA tubes processed at 96 h after blood withdrawal are not suitable for blood collection for subsequent cfDNA/ctDNA analysis as the time‐dependent increase in cfDNA concentration, resulting from leukocyte lysis, significantly affects VAF. In patient samples with low variant copy numbers, this increase in cfDNA concentration may cause variant copies to fall below the limit of detection of the dPCR assay and thus may lead to false‐negative results. Both BCT and CellSave tubes preserve cfDNA/ctDNA quality equally well up to 96 h and the used preservatives did not affect downstream cfDNA/ctDNA analyses by dPCR. Variant copy numbers and VAFs also remained stable in these tubes.

Therefore, we recommend for all future clinical studies, in which flexibility regarding the processing of blood samples is needed, to isolate plasma from blood collected in either BCT or CellSave tubes within 96 h. This will make large multicenter trials using a central processing facility feasible, and will lead to optimal quality of ctDNA for research and diagnostics.

## Funding information

This work was supported in part by KWF‐Alpe d'HuZes projects [EMCR 2014‐6340 and NKI 2014‐7080] and in part by a grant from Cancer Genomics Netherlands (CGC.nl)/Netherlands Organization for Scientific Research (NWO).

## Author contributions

LD performed plasma and cfDNA isolations, performed data analysis and co‐wrote the manuscript. NB wrote the study protocol, performed plasma isolations and co‐wrote the manuscript. JH performed the dPCR experiments and dPCR analysis. SV performd cfDNA isolations and dPCR experiments. JK, RW, SS, and MJ co‐wrote the manuscript. MPLoo performed the statistical analysis. All authors read and approved the final manuscript. JM and MPLol supervised the project and co‐wrote the manuscript.

## Supporting information


**Fig. S1**. Overview of study design.

**Fig. S2**. cfDNA concentrations for each individual patient for different preanalytical conditions.

**Fig. S3**. Correlation between variant allele frequency in tumor tissue and in ctDNA in plasma.

**Fig. S4**. Variant copy numbers for 1‐h samples.

**Table S1**. Used SNP genotyping assays
**Table S2**. Primer and probe designs for digital PCR.
