# Supplementary figures and images for: Application of circulating tumor DNA in prospective clinical oncology trials – standardization of preanalytical conditions
Source: Mol Oncol. 2017 Feb 22;11(3):295–304. doi: 10.1002/1878-0261.12037 (PMC5527445; doi:10.1002/1878-0261.12037)

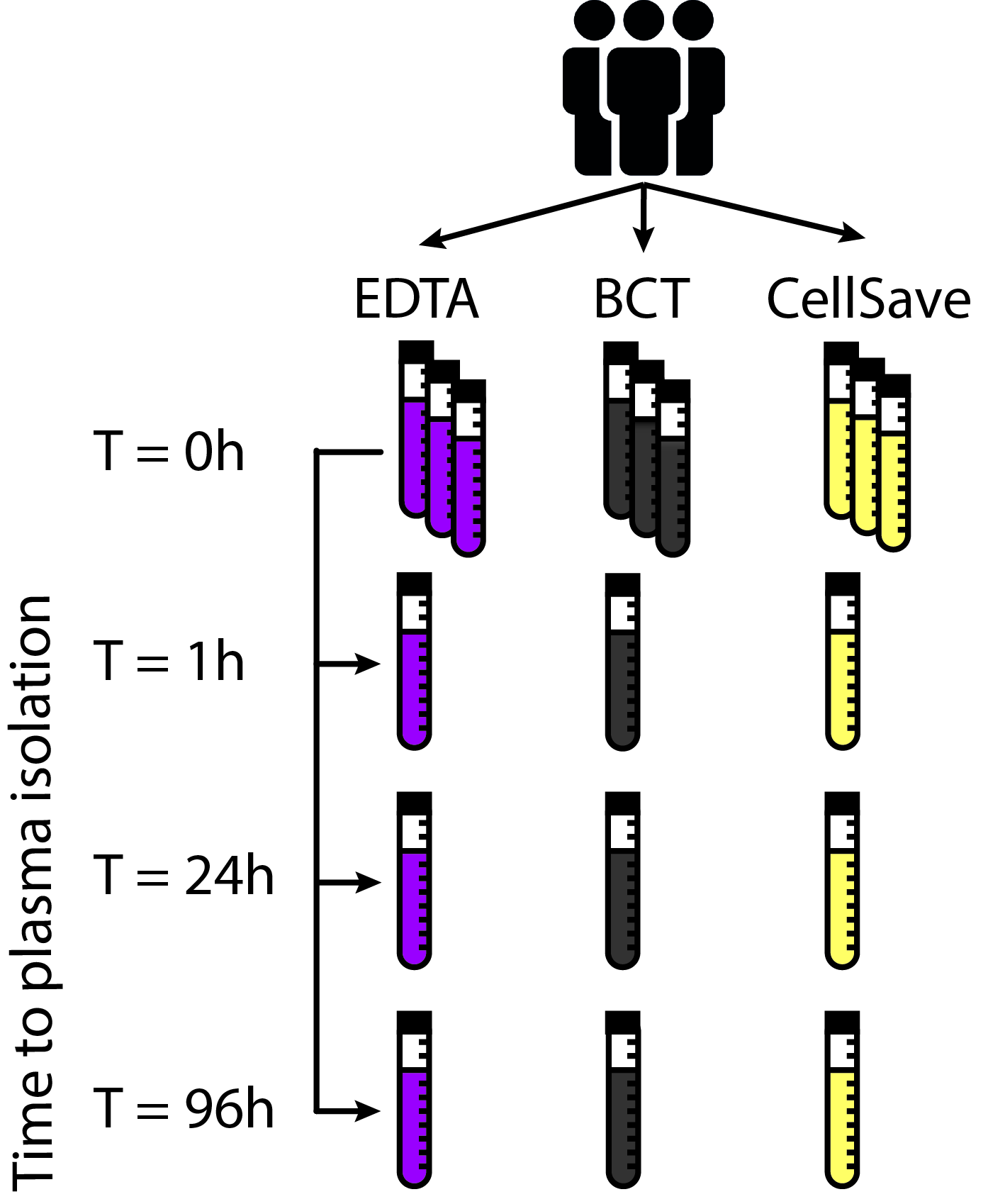

Supplement: Supplementary file 1 — Fig. S1. Overview of study design. [file MOL2-11-295-s001.tif]

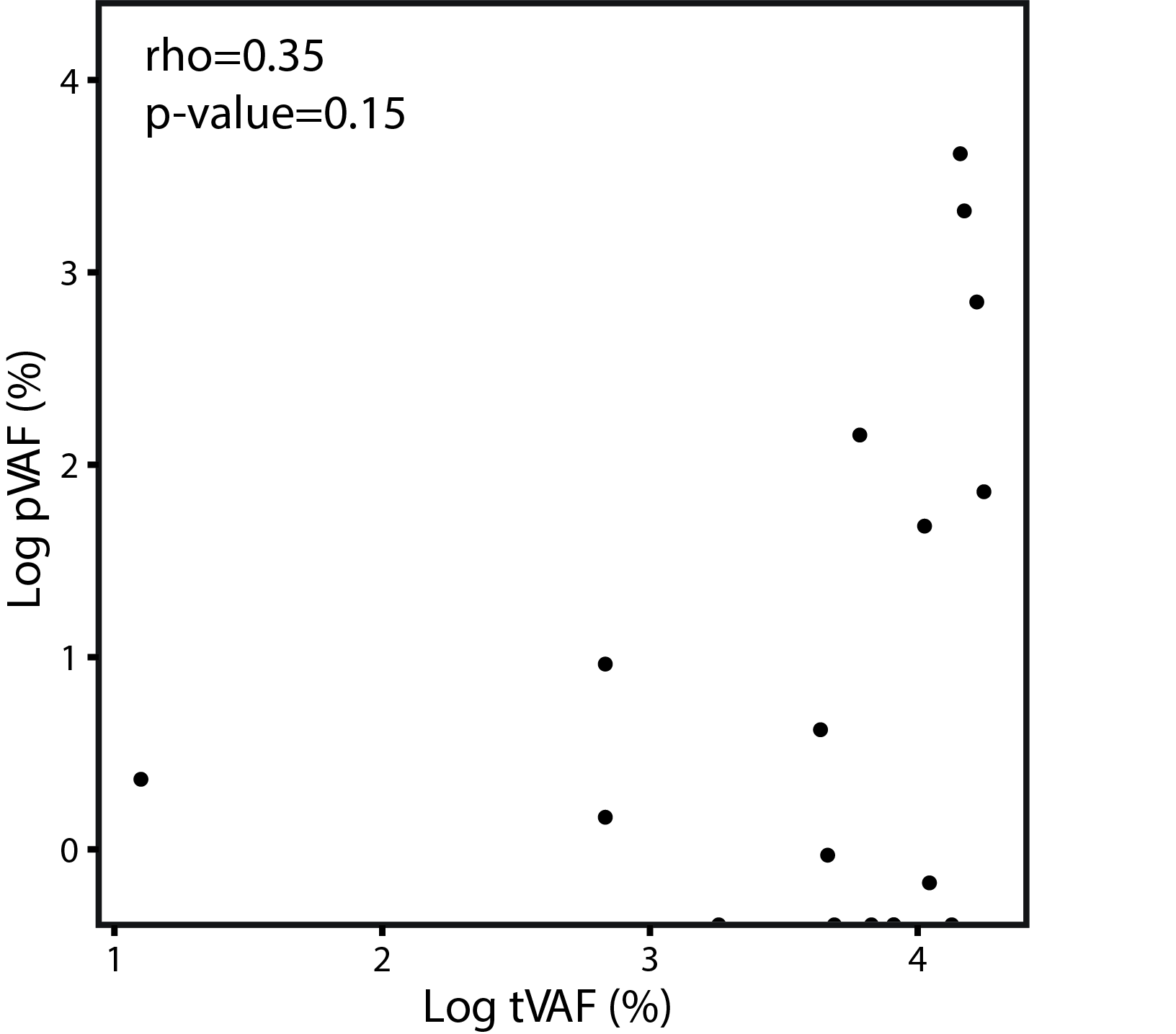

Supplement: Supplementary file 2 — Fig. S2. cfDNA concentrations for each individual patient for different preanalytical conditions. [file MOL2-11-295-s002.tif]

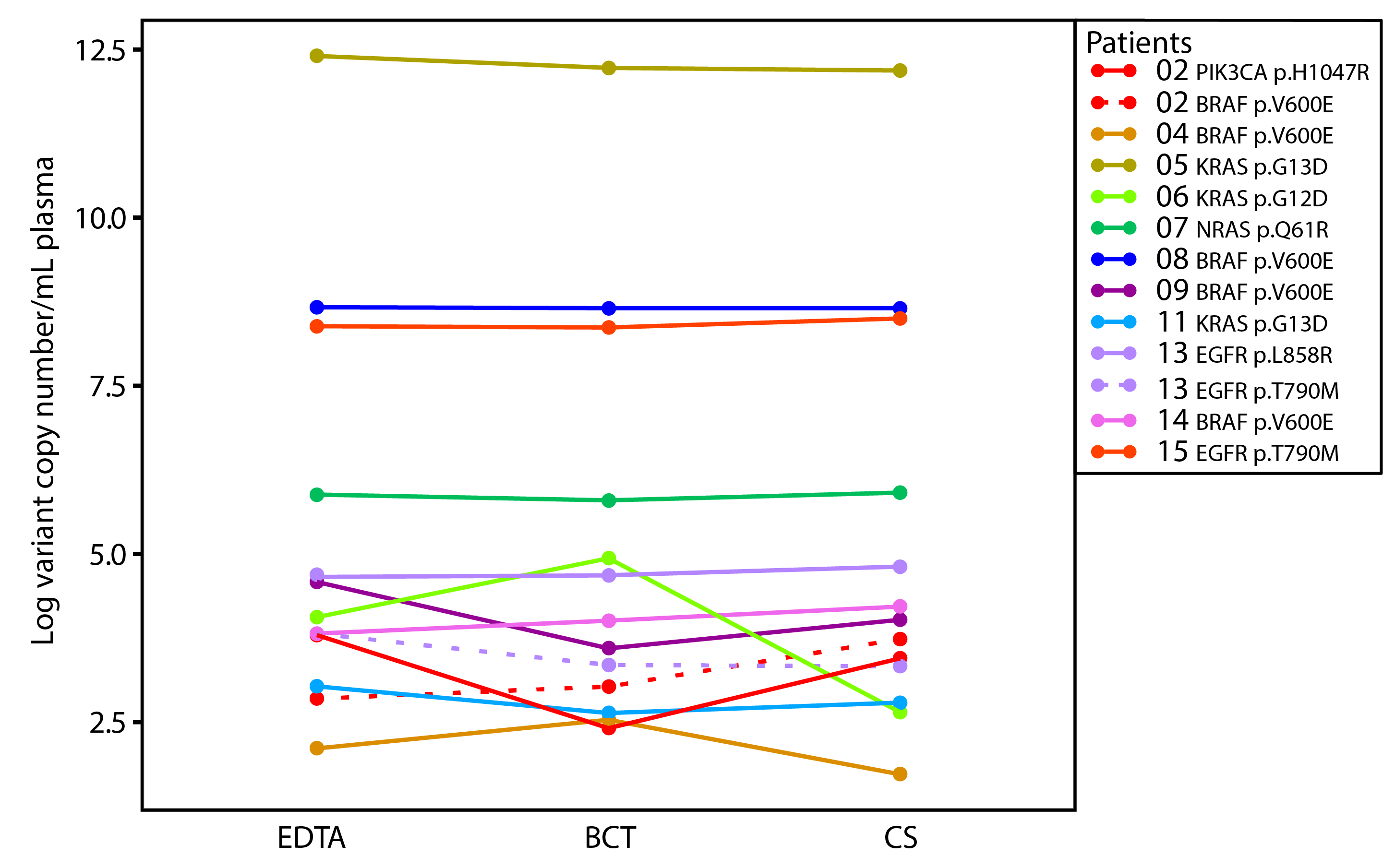

Supplement: Supplementary file 3 — Fig. S3. Correlation between variant allele frequency in tumor tissue and in ctDNA in plasma. [file MOL2-11-295-s003.tif]

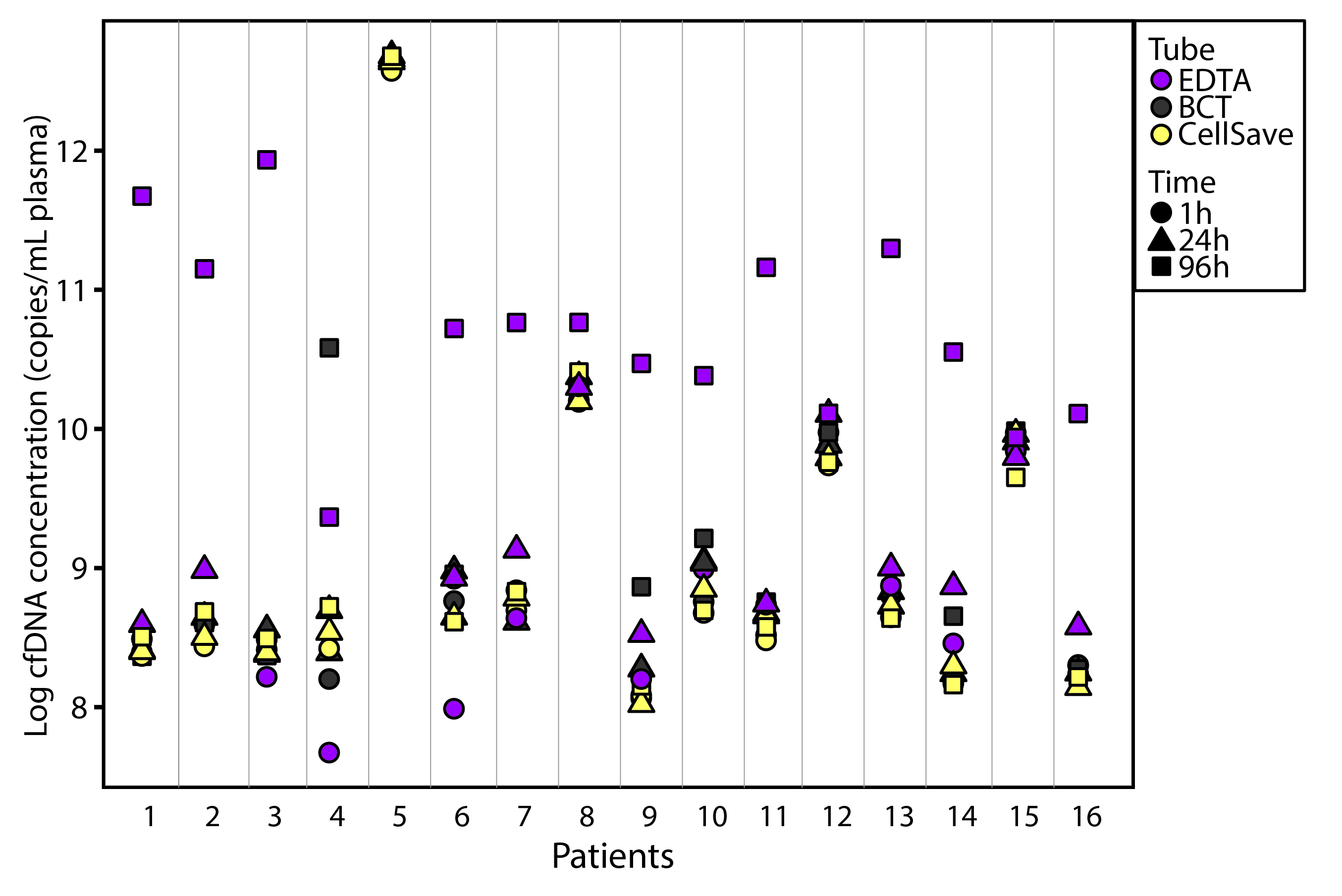

Supplement: Supplementary file 4 — Fig. S4. Variant copy numbers for 1‐h samples. [file MOL2-11-295-s004.tif]
