# Supplementary material for: Application of circulating tumor DNA in prospective clinical oncology trials – standardization of preanalytical conditions
Source: Mol Oncol. 2017 Feb 22;11(3):295–304. doi: 10.1002/1878-0261.12037 (PMC5527445; doi:10.1002/1878-0261.12037)
Supplement: Supplementary file 5 — Table S1. Used SNP genotyping assays Table S2. Primer and probe designs for digital PCR. [file MOL2-11-295-s005.docx]

**Supplemental data**

**Legends**

**Supplemental figure 1. Overview of study design.**

At time point 0h 9x10 mL blood tubes (3x EDTA; 3x BCT; 3x CellSave) were collected within a single blood draw from each recruited patient (N=16). From each type of tube plasma was isolated within 1 hour, after 24 hours and after 96 hours. Plasma was directly stored at -80°C after processing.

**Supplemental figure 2. Correlation between variant allele frequency in tumor tissue and in ctDNA in plasma.**

The log percentage of tumor tissue variant allele frequency (tVAF) on the x-axis is plotted against the log precentage of ctDNA in plasma variant allele frequency (pVAF) on the y-axis. Data points correspond to single somatic variants. Correlation was tested by Spearman's rank correlation coefficient.

**Supplemental figure 3. Variant copy numbers for 1 hour samples.**

The log variant copy numbers from 11 patients for the 1 hour samples. Data points correspond to variant copy numbers for each individual patient and assay.

**Supplemental figure 4. cfDNA concentrations for each individual patient for different pre-analytical conditions.**

Data points correspond to the log cfDNA concentrations in copies/mL plasma for each individual patient (N=16) and for the different pre-analytical conditions. Different tube types are indicated by different colors. Different time points are indicated by different symbols.

| **Supplemental table 1. Used SNP genotyping assays.** | | | | |  |
| --- | --- | --- | --- | --- | --- |
| **Assay ID** | **Assay Name** | **Gene** | **Cosmic ID** | **Amino acid change** | **Nucleotide change** |
| AH6R5PH | BRAF_476 | *BRAF* | 476 | p.V600E | c.1799T>A |
| AHRSROS | EGFR_6240 | *EGFR* | 6240 | p.T790M | c.2369C>T |
| AHRSRSV | EGFR_6224 | *EGFR* | 6224 | p.L858R | c.2573T>G |
| AH6R5PI | KRAS_521 | *KRAS* | 521 | p.G12D | c.35G>A |
| AHX1IHY | KRAS_520 | *KRAS* | 520 | p.G12V | c.35G>T |
| AHD2BW0 | KRAS_532 | *KRAS* | 532 | p.G13D | c.38G>A |
| AHQJTKH | KRAS_552 | *KRAS* | 552 | p.Q61R | c.182A>G |
| AHS1P6Q | NRAS_584 | *NRAS* | 584 | p.Q61R | c.182A>G |
| AHLJ0TP | PIK3CA_776 | *PIK3CA* | 776 | p.H1047L | c.3140 A>T |
| AHPAVCD | PIK3CA_775 | *PIK3CA* | 775 | p.H1047R | c.3140 A>G |

| **Supplemental table 2. Primer and probe designs for digital PCR.** | | | |
| --- | --- | --- | --- |
| **Gene** | **Forward primer** | **Reverse primer** | **Probe** |
| β-actin 136 bp | 5’-GCG CCG TTC CGA AAG TT-3’ | 5’- CGG CGG ATC GGC AAA -3’ | 6FAM-ACC GCC GAG ACC GCG TC-MGBNFQ |
| β-actin 420 bp | 5’-CCG CTA CCT CTT CTG GTG-3’ | 5’-GAT GCA CCA TGT CAC ACT G-3’ | VIC-CCT CCC TCC TTC CTG GCC TC-BHQ |
